# Ginsenoside RG3 and cantharidin synergistically suppress the progression of hepatocellular carcinoma via targeting the PRMT1-SREBF1 axis-mediated lipid metabolism

**DOI:** 10.1186/s12967-025-07550-8

**Published:** 2026-01-07

**Authors:** Yuehua Wang, Hengye Yuan, Yonggai Yu, Xianggang Gou, Ziyao Wang, Zhongzheng Zhou, Zezhen Wang, Wei Yan, Haisheng Wang, Jia Yan

**Affiliations:** 1 School of Basic Medicine, Inner Mongolia Medical University, Xin hua Street No. 5, Hui min District, Hohhot, Inner Mongolia 010010 China; 2 School of Life Science, Inner Mongolia University, Xin Lin Guo Le South Road 49, Yu Quan District, Hohhot, Inner Mongolia 010000 China

**Keywords:** Ginsenoside RG3, CTD, SREBF1, Lipid biosynthesis, PRMT1, HCC

## Abstract

**Background:**

Ginsenosides, such as ginsenoside RG3, demonstrate antitumor potential in hepatocellular carcinoma (HCC) and are often combined with cantharidin (CTD) in traditional Chinese medicine to achieve synergistic effects while mitigating CTD’s toxicity. However, the precise molecular mechanisms underlying this synergy remain elusive.

**Methods:**

The progression of HCC was assessed using a series of in *vitro* assays, including CCK-8 for cell viability, EdU staining for proliferation, wound healing for migration, and transwell assay for invasion. The antitumor efficacy and hepatotoxicity were assessed in animal models using mice, employing tumor volume measurement, histopathological analysis, and quantification of serum alanine aminotransferase (ALT) and aspartate aminotransferase (AST) through ELISA. To decipher the underlying synergistic mechanisms, we employed an integrated approach of network pharmacology, RNA sequencing, and molecular docking. The expression of key targets was verified by RT-qPCR and western blotting, while the direct interaction between PRMT1 and SREBF1 was confirmed by co-immunoprecipitation (Co-IP).

**Results:**

The RG3/CTD combination exhibited a potent synergistic antitumor effect, suppressing tumor proliferation, migration, and invasion more effectively than either agent alone. Mechanistically, the therapy dually modulated aberrant lipid metabolism by concurrently inhibiting the PI3K/AKT/mTOR signaling axis and PRMT1-mediated epigenetic regulation. We identified a novel direct interaction between PRMT1 and SREBF1. The binding of the CTD/RG3 complex disrupted this interaction, inhibiting PRMT1-mediated arginine methylation of SREBF1 and consequently downregulating SREBF1 expression and activity. Furthermore, RG3 significantly mitigated CTD-induced hepatotoxicity by maintaining hepatic serum ALT and AST levels, an effect likely mediated by the modulation of AKT, ACOX1, and ABCB1 pathways to reduce oxidative stress and restore metabolic homeostasis.

**Conclusions:**

Our findings establish a novel RG3-CTD regimen that concurrently enhances therapeutic efficacy and reduces hepatotoxicity through coordinated targeting of oncogenic signaling and metabolic reprogramming. This study provides a robust mechanistic foundation for the clinical translation of RG3/CTD combination therapy for HCC.

**Graphical Abstract:**

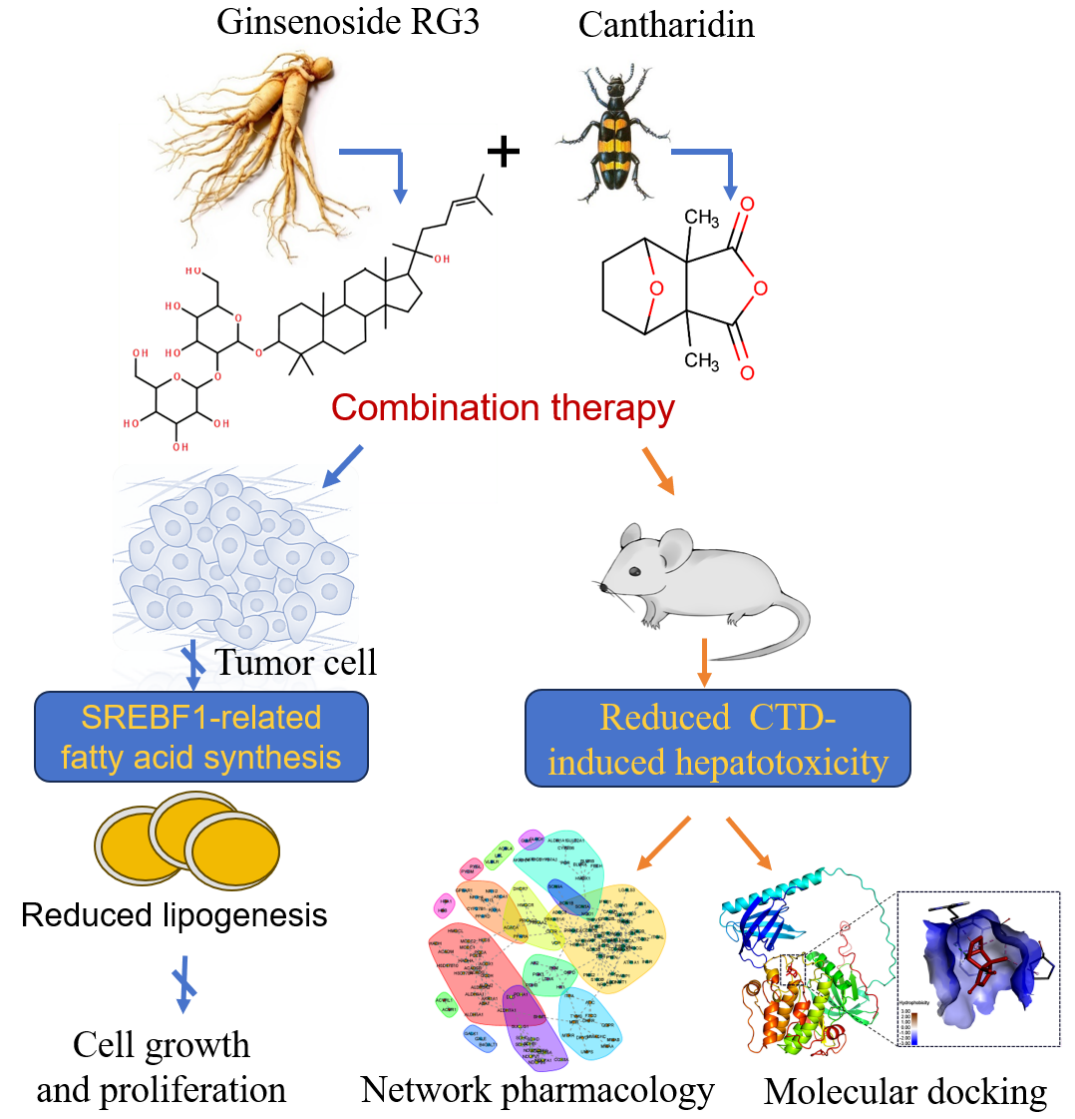

**Supplementary Information:**

The online version contains supplementary material available at 10.1186/s12967-025-07550-8.

## Introduction

CTD, a natural terpenoid from the blister beetle *Mylabris phalerata Pallas*, has been used in traditional Chinese medicine for centuries and recently recognized for its anti-cancer effects, particularly in HCC [1–3]. It inhibits tumor growth by inducing cell cycle arrest, apoptosis, modulating autophagy, disrupting DNA repair, and regulating oncogenic pathways [4–8]. Our study also highlights its multi-target action in HCC through autophagy regulation, immune microenvironment modulation, fatty acid metabolism reprogramming, and targeting the EZH2/H3K27me3 cell cycle axis [9, 10]. However, the clinical translation of CTD remains constrained by its dose-dependent hepatotoxicity, necessitating innovative strategies to preserve therapeutic efficacy while minimizing hepatic damage.

These challenges have spurred interest in developing rational combination therapies. Systematic analysis of traditional CTD-containing formulations reveals a notable co-occurrence with *Panax* ginseng components across multiple therapeutic combinations, such as aidi injection, and disodium cantharidinate/vitamin B₆ injection [11]. Ginsenosides from Panax ginseng demonstrate dual pharmacological advantages, synergistic enhancement of chemotherapeutic efficacy through tumor microenvironment modulation and hepatoprotective properties [11]. Building on these insights, we propose that strategic combination of ginsenosides with CTD may achieve dual therapeutic optimization-amplifying anti-HCC effects while counteracting CTD-induced hepatotoxicity, potentially overcoming current limitations in monotherapy approaches.

Ginsenoside RG3, a bioactive saponin from *Panax* ginseng, features a tetracyclic hydrophobic structure with multi-sugar modifications that confer broad pharmacological properties [11]. Emerging evidence highlights its multi-mechanistic anti-HCC effects. RG3 was been demonstrated a remarkable therapeutic potential by impeding cell proliferation, enhancing immune responses, reducing angiogenesis and metastasis, and promoting cell apoptosis [12, 13]. RG3 modulates several critical signaling pathways, including PI3K/AKT, NHE1/EGF-EGFR-ERK1/2-HIF-1α, IFN-γ/IL-2, ROS/LC3 II, ARHGAP9, and PCNA/cyclin D1 in HCC [14–17]. Notably, RG3 synergizes with sorafenib to dual-regulate PI3K/AKT/STAT3 signaling and HK2-mediated glycolysis, simultaneously upregulating PTEN while downregulating AKT [18, 19]. This combination demonstrates enhanced efficacy in overcoming drug resistance through ROS/STAT3 axis modulation, establishing RG3 as a promising therapeutic adjuvant for HCC treatment [20].

Ginsenosides have emerged as prominent hepatoprotective agents, with ginsenoside RG3 standing out due to its natural abundance and multi-therapeutic effects across liver pathologies [21]. A growing body of literature has elucidated the multifaceted hepatoprotective properties of RG3, demonstrating its efficacy in ameliorating diverse hepatic pathologies, including viral hepatitis, acute liver injury, nonalcoholic fatty liver disease (NAFLD), hepatic fibrosis, and HCC [22, 23]. Therefore, we speculated that RG3 presents a rational strategy for addressing CTD-associated hepatotoxicity in HCC combination therapies.

The molecular mechanisms underlying RG3-mediated hepatoprotection are multifaceted, involving the modulation of inflammatory cascades and oxidative stress pathways. Specifically, RG3 has been shown to exert protective effects against acetaminophen-induced hepatotoxicity through the simultaneous regulation of inflammatory mediators and oxidative stress markers [24]. Emerging evidence suggests that RG3 orchestrates a complex regulatory network involving the upregulation of TUG1 expression, suppression of miR-200a-3p, and activation of the SIRT1/AMPK signaling axis, collectively contributing to the attenuation of sepsis-induced hepatic damage [25, 26]. These molecular insights not only underscore the therapeutic potential of RG3 in mitigating drug-induced liver injury but also provide a compelling rationale for its application as an adjunctive therapy in CTD-based anticancer regimens.

While RG3-CTD co-therapy demonstrates therapeutic promise for HCC, two fundamental questions remain unresolved, the molecular basis of their synergistic antitumor effects, particularly concerning metabolic reprogramming in hepatocarcinogenesis; the feasibility of concurrent hepatoprotection and tumor suppression. This study addresses these knowledge gaps through a dual investigative approach. First, we employ network pharmacology to decode combination mechanisms, with focused interrogation of fatty acid metabolism modulation - a pivotal HCC driver. Second, we mechanistically dissect RG3’s capacity to neutralize CTD-induced hepatic damage. Our multimodal validation reveals this combination operates as a dual-axis therapeutic system, potentiating anticancer effects through metabolic pathway disruption while establishing hepatoprotective safeguards, thereby creating a translational bridge between mechanistic understanding and clinical application in HCC management.

## Materials and methods

### Reagents and cells

Cantharidin (Sigma-Aldrich, purity ≥ 98%) and ginsenoside RG3 (Beijing Solarbio Science & Technology, China, purity ≥ 98%) were used. HepG2, MHCC97-H and Hepa1-6 cells (Guangzhou Saiye, China) were cultured in DMEM (Gibco, Thermo Fisher Scientific, USA) with 10% FBS, 100 U/mL penicillin, and 100 µg/mL streptomycin at 37 °C, 5% CO₂.

### Cell proliferation assay

#### CCK8 assay

Cell viability was determined by CCK-8 assay (Yeasen, Shanghai, China), and was performed as previously described [10].

#### EdU staining assay

Cells in 12-well plates were incubated with diluted EdU647 solution (Beijing Solarbio Science & Technology, China) for 2 h, then stained with Hoechst 33,342. After three washes, the proportion of EdU-positive cells was quantified. All experiments were carried out with three biological replicates.

### Wound healing assay

The wound healing assay was conducted, and a scratch was generated using a pipette tip. Subsequently, the cells were incubated in serum-free medium. The progression of wound closure was meticulously monitored at 24 h, and the residual cell-free area was measured after capturing images. Each independent experiment was replicated at least three times.

### Cell invasion assay

HCC cells were resuspended in serum-free medium at a density of 2 × 10⁵ cells/mL. A volume of 150 µL of the cell suspension was seeded into the upper chamber of a Transwell insert, while 700 µL of medium containing 10% FBS was added to the lower chamber as a chemoattractant. After 24 h of incubation, the cells on the membrane were fixed with methanol for 30 min at room temperature, and then stained with Giemsa solution for 15–30 min. Non-invaded cells on the upper surface of the membrane were carefully removed with a cotton swab. The membrane was then air-dried, mounted with neutral gum, and examined under a microscope. Each experiment was independently repeated at least three times.

### Lipid droplets staining assay

Cultured cells were passaged onto 12-well plate cell crawls, fixed with 4% paraformaldehyde solution (Biosharp, China), washed three times in 1×PBS, and then stained with 2 µg/mL nile red solution (HY-D0718, MCE, USA) for 10 min. The samples were then washed three times with PBS, and then stained with DAPI for 5 min, washed three times and then the images were captured using confocal microscopy.

### Construction of PPI network

The PPI networks were constructed utilizing the STRING database and subsequently visualized using Cytoscape software.

### Target clustering and enrichment analysis

GO, KEGG, and GSEA was utilized for functional and pathway analysis using the cluster Profiler packages available on the R platform. Cytoscape software was employed to create a comprehensive target-pathway network.

### Establishment of animal model

All animal experiments were conducted with the approval of the Animal Ethics Committee of Inner Mongolia Medical University and adhered to the guidelines set forth by the Basic & Clinical Pharmacology & Toxicology policy for experimental and clinical studies.

#### Toxicity experiments on animals

The 6-week-old C57BL/6 mice were obtained from Vital River Animal Technology. Following one-week acclimatization period, Mice with similar body weights (22.5 ± 0.5 g) were randomly divided into four groups, each consisting of 5 mice (*n* = 5): control group (saline solution), CTD (1.5 mg/kg), RG3 (15 mg/kg), and CTD (1.5 mg/kg)/ RG3 (15 mg/kg). Each group underwent treatment for two weeks. At the conclusion of the treatment period, blood and tissues were obtained for subsequent pathological analysis. Their liver samples were fixed in 4% paraformaldehyde for H&E staining.

#### HCC cell-bearing model

The female BALB/c nude mice (4 weeks old, 20–23 g) were obtained from Vital River Animal Technology. Then Hepa1-6 cells (5 × 10^6^) were subcutaneously injected into armpits of nude mice. Mice with successful tumor-bearing tumors of similar size were randomly divided into four groups with 5 mice in each group (*n* = 5), Model group (saline solution), CTD treatment group (1.0 mg/kg), RG3 treatment group (10.0 mg/kg), combination treatment group (CTD 1.0 mg/kg + RG3 10.0 mg/kg). After two weeks, the mice were sacrificed for analysis. Tumor volume was measured every three days using the formula: volume = length × width^2^ × 0.5. Subsequently, IHC was performed to detect the expression of SREBF1, FASN, and Ki67 proteins level in the tumor samples. The antibodies were procured from Abcam Company in the USA.

### Biochemical assays

To evaluate liver function, the serum of mice model in each group was collected, and the ALT and AST levels were measured to evaluate liver function using biochemical kit (Beijing Solarbio Science & Technology, China). The ALT and AST levels were measured thrice for each group (*n* = 3).

### Hematoxylin and Eosin (H&E) staining

The tissues from each group (*n* = 5) were fixed in 4% paraformaldehyde, dehydrated, paraffin-embedded, and sectioned into 4 μm slices. After xylene dewaxing and graded ethanol rehydration, sections were hematoxylin-stained (nuclei blue), differentiated in HCl-alcohol (30 s), eosin-stained (cytoplasm/matrix pink, 2 min), and imaged using a digital scanner microscope.

### Real-time-PCR

Total RNA was extracted using Takara RNA Plus reagent, reverse-transcribed with PrimeScript RT Kit (gDNA Eraser), and quantified via SYBR Premix Ex Taq II (Takara, China) on a Bio-Rad CFX6 Thermal Cycler. GAPDH was used as the internal control, with target gene expression normalized by 2 (2-ΔΔCt). The experiment was performed in triplicate, and the average value was calculated.

### Western blotting

Cells are collected and total proteins are obtained. The protein concentrations were then determined using the Bio-Rad Protein Assay Kit. SDS-PAGE (Sodium Dodecyl Sulfate Polyacrylamide Eel Electrophoresis) was conducted, followed by the transfer of proteins onto PVDF (Polyvinylidene Difluoride) membranes (Merck & Millipore, USA). The membranes were then incubated with primary antibodies overnight, followed by a 1-hour incubation with secondary antibodies. For visualization, an ECL (Enhanced Chemiluminescence) Kit was utilized. The band densitometry analysis was performed using Image J software. The experiment was performed in triplicate, and the average value was calculated. The antibodies, specifically SREBF1, SREBF2, FASN, SCD1, and PCSK9, were procured from Abcam Company in the USA.

### Molecular docking

Target protein 3D structures were obtained from the Protein Data Bank. Autodock Tools removed water molecules, co-crystallized ligands, and heteroatoms. Ligands were prepared using ChemBioDraw 3D for energy-minimized 3D structure generation. Compounds were processed in AutoDock Tools 1.5.6 (rotatable bonds set as default) and saved as PDBQT files. Docking analysis and visualization were performed with Autodock Vina 1.1.2 and Discovery Studio 3.5.

### Cellular thermal shift assay (CETSA)

Logarithmically growing HCC cells were seeded into T75 flasks. Upon reaching 90% confluency, the cells were divided into two groups and treated with either 0.1% (v/v) DMSO or CTD/RG3, respectively. After 24 h of treatment, the cells were harvested by centrifugation and washed with PBS. The cell pellets were resuspended and incubated on a shaking platform at 37 °C for 1 h. Each sample was then aliquoted into six equal portions and subjected to a temperature gradient ranging from 37 °C to 67 °C in increments of 6 °C using a PCR instrument. The samples were maintained at each target temperature for 10 min. Following heat treatment, three freeze-thaw cycles were performed using liquid nitrogen. After centrifugation, the supernatants were collected and mixed with 5× loading buffer. The mixtures were then boiled at 100 °C for 5 min. Finally, Western blot analysis was conducted to evaluate the expression levels of the target protein.

### Immunofluorescence assay

Tissue specimens underwent fixation in 4% methanol-free formaldehyde. Permeabilization (0.25% Triton X-100/PBS, 10 min) and blocking (10% FBS/PBS, 1 h) preceded sequential incubations: primary antibodies (4 °C overnight), then species-matched Alexa Fluor secondary antibodies. Nuclear counterstaining was performed using DAPI, and fluorescent images were acquired using confocal.

### Co-immunoprecipitation (Co-IP) assay

Cells were lysed in RIPA buffer with protease/phosphatase inhibitors, centrifuged, and precleared with Protein A/G beads (Thermo Fisher Scientific, USA). Lysates (500 µg) were incubated with PRMT1 /IgG antibody (Abcam, USA) (2 µg, 4 °C overnight), then Protein A/G beads (20 µL, 2 h). Immunocomplexes were washed by lysis buffer, eluted in Laemmli buffer, and analyzed by SDS-PAGE/immunoblotting.

### Statistical analysis

Statistical analyses were performed using GraphPad Prism 8.0. One-way or two-way ANOVA, followed by Dunnett’s test, was used for comparisons among multiple groups. Data are presented as mean ± standard error of the mean (SEM). A *p*-value of less than 0.05 was considered statistically significant. Significance levels are denoted as follows: *p* < 0.05 (*), *p* < 0.01 (**), *p* < 0.001 (***), *p* < 0.0001 (****), or ns (not significant). All experiments were performed with at least three independent replicates.

## Results

### Synergistic inhibition of cell proliferation and metastasis through combination therapy of RG3 and CTD in HCC

**Fig. 1:**
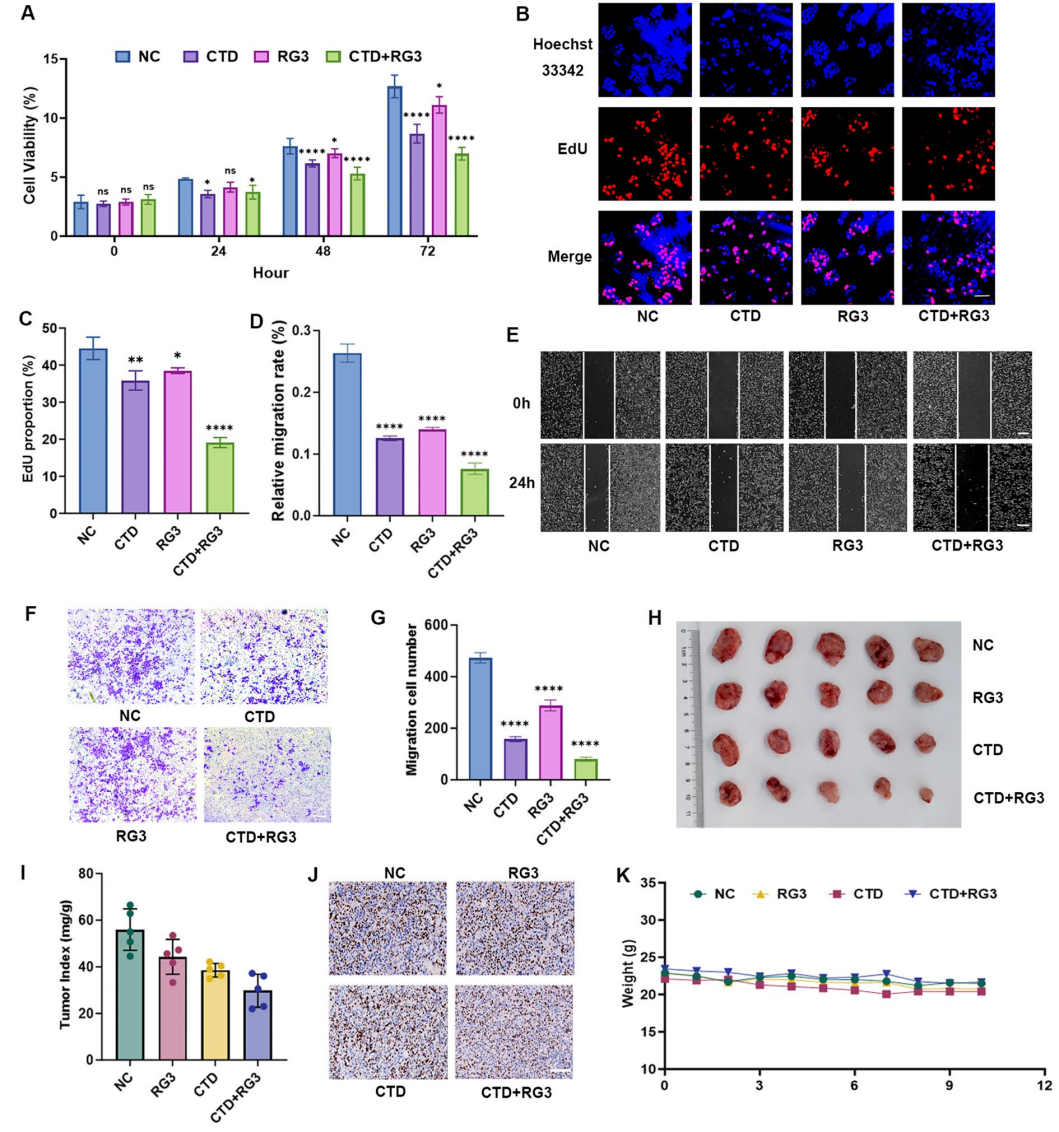
RG3 and CTD inhibit HCC cell growth and migration in vitro and in vivo. (**A**) RG3/CTD dose-dependently suppressed MHCC97-H proliferation, assessed by CCK-8 assay. Two-way ANOVA, Sidak’s multiple comparison tests (comparison between the treatment groups at each timepoint. (**B**, **C**) DNA replication inhibition via EdU assay in MHCC97-H cells. **(B**) representative confocal micrographs and (**C**) quantitative analysis. Nuclei counterstained with DAPI (blue), EdU+ cells (red). The scale bar represents 20 μm; magnification 20×. (**D**, **E**) Wound closure rates in scratch assays for MHCC97-H cells. (**F**, **G**) Migratory cell visualization and statistical analysis from transwell chambers. The scale bars represent 100 µm. Data are represented as mean ± SEM of three independent experiments; and *p < 0.05; **p < 0.01; ****p < 0.0001 vs. the control group (Student’s t-test). (**H**, **I**) RG3 and CTD represses the tumor cell growth in vivo. The tumor volume (**H**) and tumor weight index, the ratio of tumor weight to the body weight of mice (**I**) were shown (n = 5; one-way ANOVA on ranks). (**J**) IHC staining of tumor tissues excised from mice demonstrates that the expression level of Ki67 protein were significantly downregulated following treatment with RG3 and CTD. magnification: 40×. Each experimental group consisted of 5 mice (n = 5). (**K**) Graph of body weight changes in mice (n = 5; one-way ANOVA on ranks)

To elucidate the combinatorial effects of RG3 and CTD on the progression of HCC, we systematically evaluated their impact on cell proliferation, migration, and invasion. CCK-8 assays showed a greater reduction of cell viability with combination treatment compared to CTD or RG3 monotherapy in HCC cells, including MHCC97-H and HepG2 cells (Fig. 1A, S1A). Moreover, EdU staining results indicated that the combined treatment groups showed a decrease of DNA-replicating cells (Fig. 1B, C and S1B, C). Furthermore, wound healing assay revealed a substantial impairment in cellular migration with combination group (Fig. 1D, E and S1D, E). The transwell invasion assay indicated a remarkable decrease in invasive capacity with the combination therapy relative to untreated HCC cells (Fig. 1F, G and S1 F, G). The combinatorial treatment exhibited superior efficacy in inhibiting HCC cell proliferation and metastasis compared to monotherapy with either agent alone in *vitro*.

Furthermore, in *vivo* validation using allograft models also confirmed these findings, combinatorial treatment significantly attenuated tumor progression compared to vehicle controls with a reduction in final tumor volume compared to monotherapy (Fig. 1H, I). Moreover, we performed immunohistochemistry analysis of tumor tissues to assess the expression of Ki67-positive cells, which are widely recognized as markers of cellular proliferation. The results also indicated a reduction in tumor tissue following combinatorial treatment (Fig. 1J). Notably, body weight monitoring demonstrated comparable weight loss trajectories across all treatment groups post-implantation, with no statistically significant differences in maximum weight loss between cohorts (Fig. 1K). Taken together, CTD and RG3 exhibited a synergistic effect in inhibiting the progression of HCC.

### Integrated network pharmacology elucidation the mechanism of RG3 and CTD combination therapy in HCC

**Fig. 2:**
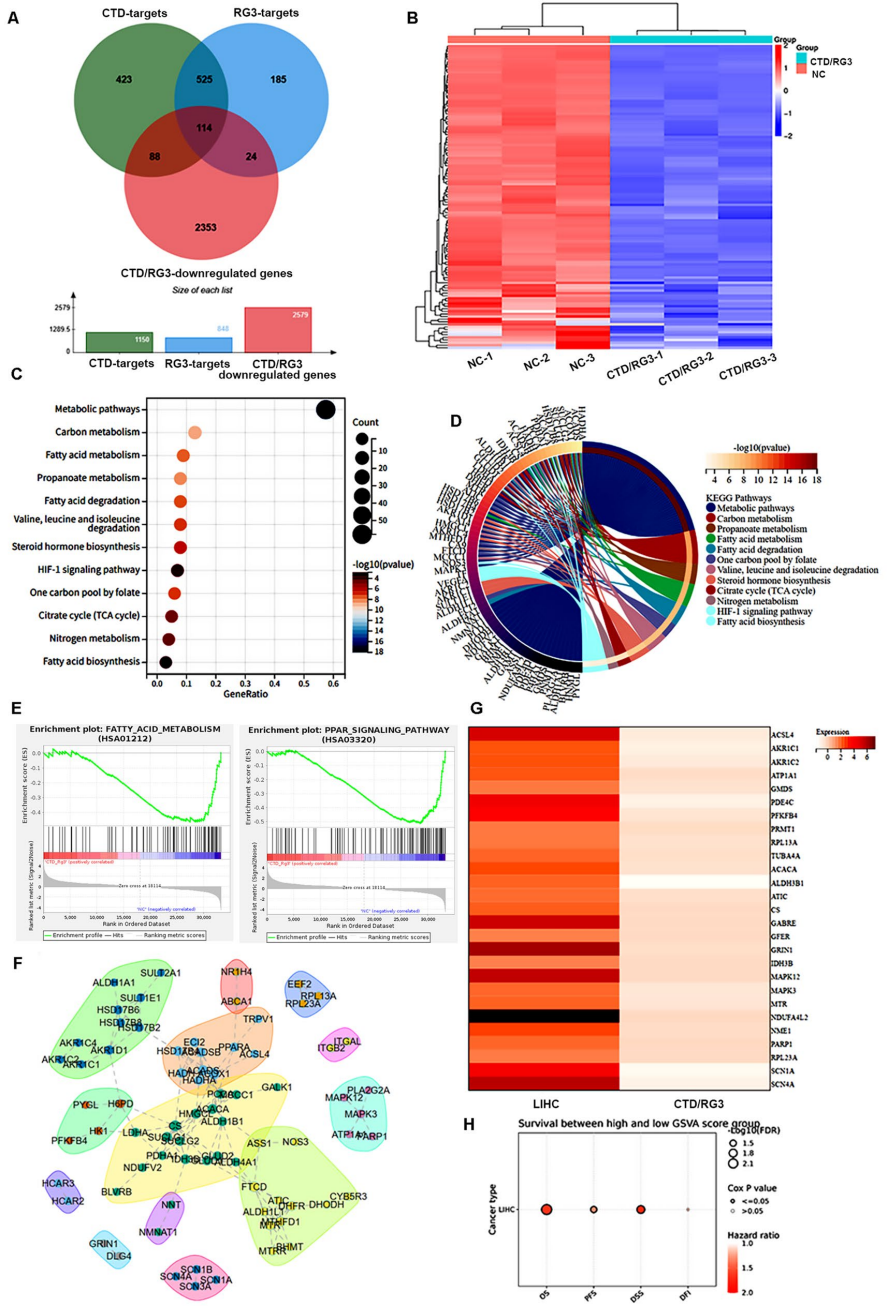
Identification of potential targets for the combination therapy of RG3 and CTD in HCC. (**A**) Venn diagram illustrating the overlap of targets associated with CTD and RG3, and the CTD/RG3 related differentially expressed genes in HCC cells. (**B**) Heatmap visualization of expression patterns for key target genes across experimental groups. (**C**) KEGG analysis of these hub targets of CTD and RG3. (**D**) The relationship between the predicted targets and their associated pathways is depicted in a circular diagram based on KEGG enrichment. (**E**) Gene set enrichment analysis of treatment-related DEGs showing enriched biological processes and pathways. (**F**) PPI network of potential therapeutic targets constructed using STRING database. (**G**) Schematic overview of CTD/RG3 combination therapy targets in HCC, with accompanying heatmaps comparing, (left) gene expression profiles in clinical HCC samples vs adjacent tissues, and (right) expression changes post-combination treatment in HCC cells. (**H**) Correlation heatmaps illustrating prognostic significance of target genes in HCC, with color gradients representing hazard ratios from Cox regression analysis

To elucidate the synergistic anti-HCC mechanisms of RG3 and CTD, we integrated network pharmacology with transcriptomic profiling of RNA-sequenced HCC cells treated with these compounds. Database mining (HERB/ETCM) identified 848 RG3 and 1,150 CTD targets (Fig. 2A). Intersection analysis with RG3 and CTD-induced downregulated DEGs revealed 114 overlapping therapeutic targets via Venn analysis (Fig. 2A). The expression of these target genes was down regulated in co-treatment of HCC cells with RG3 and CTD, and their gene expression profile was shown in the heatmap (Fig. 2B). Further KEGG enrichment analysis linked these targets to metabolic pathways, carbon metabolism, and fatty acid metabolism/degradation (Fig. 2C, D). Moreover, GSEA results also confirmed that these genes were significantly associated with fatty acid metabolism pathways (Fig. 2E). Subsequently, PPI network analysis (STRING database; confidence score > 0.75) was constructed and organized these targets into six functional hub modules (Fig. 2F).

To identify therapeutic targets of the CTD/RG3 combination in HCC, we analyzed the expression of 114 candidate genes in patients with LIHC based on the TCGA data. Comparative profiling revealed 27 genes significantly upregulated in tumor versus adjacent tissues of LIHC patients. Notably, combination therapy significantly downregulated this 27-gene signature in HepG2 cells based on RNA-seq data. (Fig. 2G). Prognostic analysis demonstrated their significant association with poor clinical outcomes (Fig. 2H), supporting their utility as key therapeutic targets for this combinatorial strategy.

### RG3 and CTD synergistically suppress SREBF1-FASN/SCD1 axis-mediated lipid metabolism in HCC

**Fig. 3:**
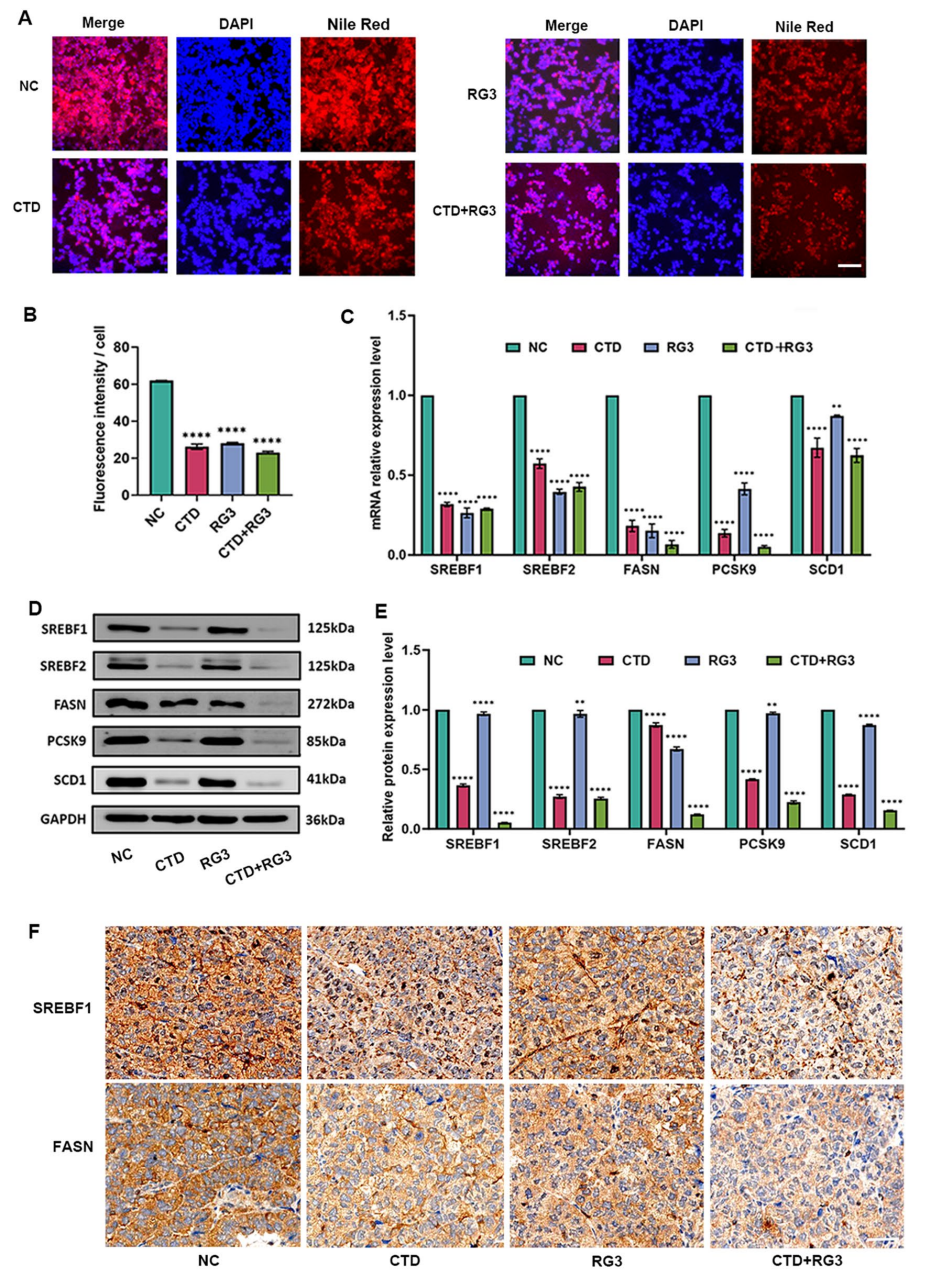
CTD and RG3 suppress fatty-acid metabolism via SREBF1 pathways in HCC. (**A**) Nile red staining reveals lipid droplet accumulation in HCC cells treated with RG3, CTD, and their combination. The scale bar represents 20 μm; magnification 20×. (**B**) The accumulation of lipid droplets was decreased in the treated cells compared to control cells. (**C**) Analysis of mRNA level, and (**D**, **E**) protein level of lipogenesis-related genes, including SREBF1, SREBF2, FASN, SCD1, and PCSK9 in HCC cells treated with RG3, CTD, and their combination. The Data are represented as mean ± SEM of three independent experiments; The data are from three independent experiments (n = 3); ns *p* > 0.05; **p < 0.01; ****p < 0.0001 vs. the control group. (**F**) IHC staining of tumor tissues excised from mice demonstrates that the expression level of SREBF1 and FASN proteins were significantly downregulated following treatment with RG3 and CTD. Magnification 40×. Each experimental group consisted of 5 mice (n = 5)

Given the KEGG/GSEA enrichment in fatty acid metabolism, we investigated the combinatorial effects of RG3 and CTD on lipid metabolic remodeling in HCC. As illustrated in Fig. 3 and S2, the extent of Nile red staining in the RG3 and CTD combination group was significantly reduced compared to the control group in HCC cell lines, HepG2 and MHCC97-H (Fig. 3A, B and Figure S2A, B). Furthermore, molecular characterization of pathways associated with fatty acid synthesis revealed that RG3 and CTD markedly downregulated the expression of key lipogenic enzymes at both transcriptional and translational levels. The combination treatment led to a reduction in mRNA expression levels of *SREBF1*, *SREBF2*, *FASN*, *PCSK9*, and *SCD1* (Fig. 3C and Figure S2C). Additionally, western blot analysis corroborated these findings at the protein level (Figs. 3D, E and Figure S2D, E). Additionally, IHC results revealed a decrease in the expression of proteins associated with fatty acid synthesis, such as SREBF1 and FASN, in tumor tissues after treatment with either CTD or RG3. The combination treatment led to a more pronounced reduction compared to monotherapy (Fig. 3F). These findings indicate that RG3 and CTD synergistically inhibit the SREBF1-FASN/SCD1 axis to disrupt lipid metabolism in HCC.

### RG3 and CTD synergistically regulates SREBF1-mediated lipid metabolism via PI3K/AKT/mTOR signaling pathway

**Fig. 4:**
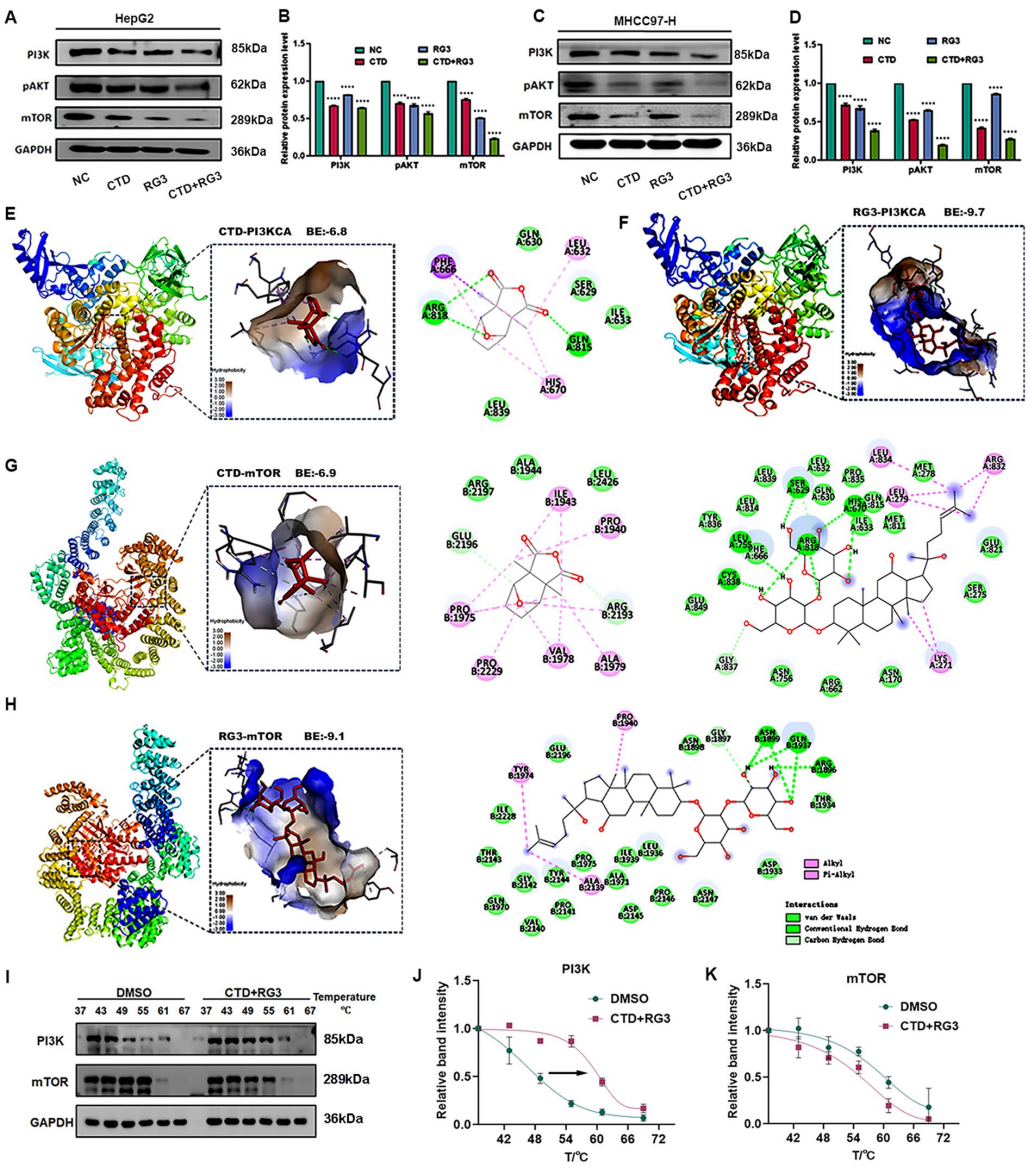
CTD and RG3 suppress PI3K-mTOR pathways in HCC. (**A**–**D**) RG3 and CTD modulate the PI3K/AKT/mTOR pathway in HCC cells. Western blot analysis of key pathway proteins in cells treated with RG3, CTD, or their combination. (**A**, **B**) The protein expression in HepG2 cells; (**C**, **D**) The protein expression in MHCC97-H cells. Data are presented as mean ± SEM of three independent experiments; ****p < 0.0001 versus the control group. (**E**–**H**) Molecular docking predicts direct binding of RG3 and CTD to pathway components. Binding modes of (**E**, **F**) RG3 or CTD with PI3KCA, and (**G**, **H**) with mTOR. (**I**–**K**) CETSA validates direct target engagement in cells. (**I**) Western blot results from the CETSA. Quantitative analysis of thermal stability for (**J**) PI3K and (**K**) mTOR proteins upon ligand binding (n = 3). Data are presented as mean ± SEM of three independent experiments

Given the established role of the PI3K/AKT/mTOR axis in metabolic regulation, we investigated its involvement in RG3/CTD-mediated lipid metabolic reprogramming. The quantitative analysis revealed a significant downregulation of the proteins PI3K, pAkt, and mTOR in HCC cells following combination treatment (Fig. 4A-D). To elucidate structural basis of pathway inhibition, we performed molecular docking simulations with PI3K catalytic subunit (PIK3CA) and mTOR kinase domains. RG3 exhibited strong binding affinities to both targets with calculated values of -9.7 kcal/mol (PI3KCA) and −9.1 kcal/mol (mTOR), while CTD showed moderate interactions at -6.8 kcal/mol and −6.9 kcal/mol, respectively (Fig. 4E-H).

To further validate the direct targets of RG3 and CTD, we employed the cellular thermal shift assay to assess their binding affinity toward PI3KCA and mTOR in HCC cells. The thermal shift curves demonstrated a rightward shift in the melting profiles of PI3KCA following treatment with CTD/RG3. PI3K activity in the DMSO group declined with increasing temperature, it was markedly preserved in the CTD/RG3-treated group (Fig. 4I, J). However, there is no significant shift in the thermal stability curve of mTOR (Fig. 4I, K). These findings indicate that PI3K serves as a direct target of RG3 and CTD, suggesting that these compounds directly target the PI3K protein to disrupt lipid synthesis and suppress HCC progression.

### RG3 and CTD regulate SREBF1-related lipid metabolism by targeting PRMT1

**Fig. 5:**
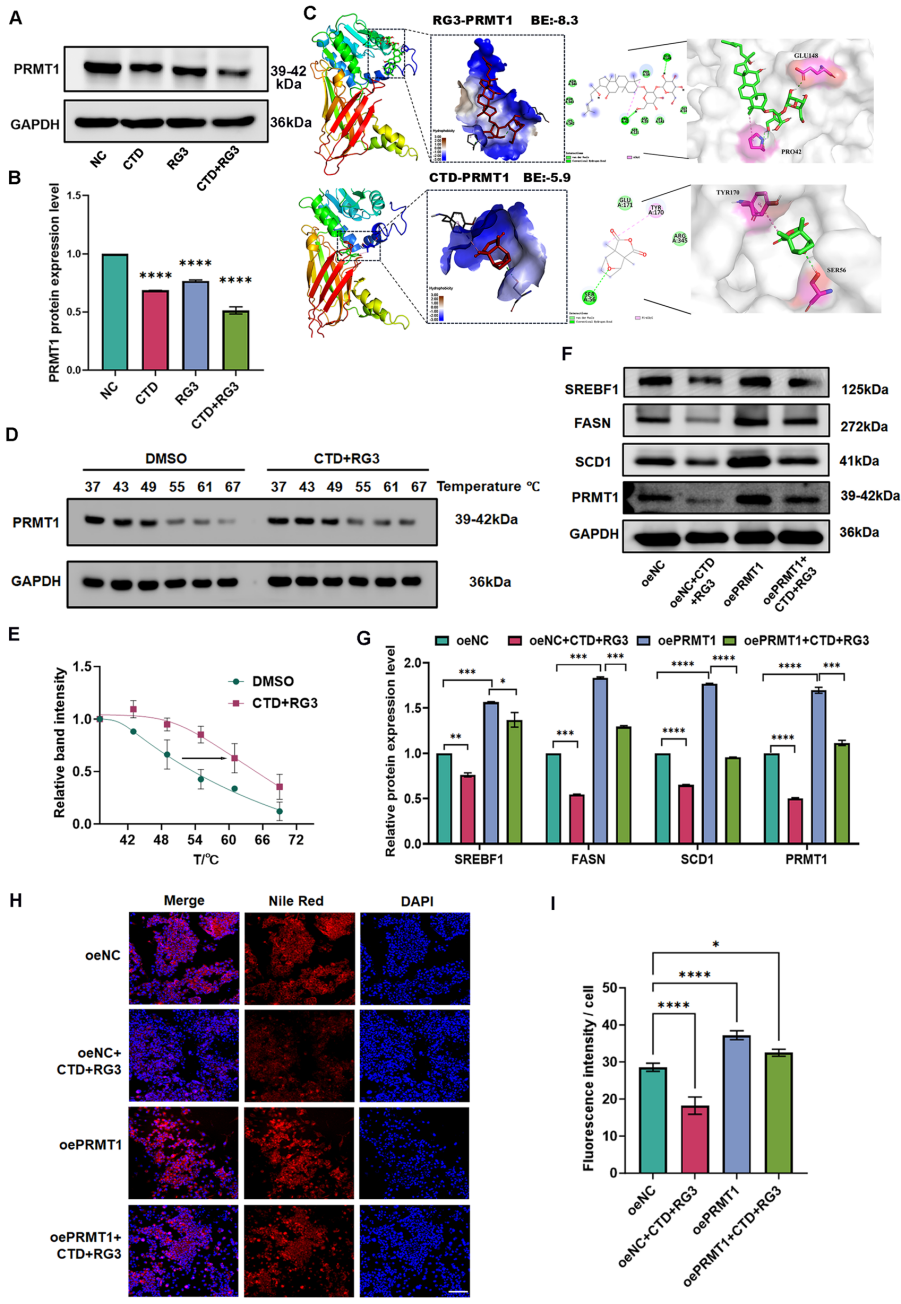
RG3 and CTD regulate PRMT1 to affect SREBF1-associated fatty-acid metabolism. (**A**, **B**) The protein level of PRMT1 in HCC cells treated with RG3, CTD, and their combination. (**A**) Western blot results; (**B**) Results of protein grayscale analysis. Data are presented as mean ± SEM of three independent experiments; ****p < 0.0001 vs. the control group. (**C**) Molecular docking diagram of PRMT1 and RG3 or CTD. (**D**, **E**) CESTA analysis between RG3/CTD and PRMT1. (**D**) Western blot results from the CETSA test; (**E**) Results of protein grayscale analysis. (**F**, **G**) Analysis the protein expression of fatty acid metabolism synthesis related pathway proteins, including SREBF1, FASN, SCD1 in HCC cells treated with RG3 and CTD combination and over expression PRMT1 protein. (**H**, **I**) The accumulation of lipid droplets was decreased in the HCC cells, CTD and RG3 combined treatment group, PRMT1 overexpression group, and simultaneous CTD and RG3 treatment with overexpression of PRMT1. (**H**) Fluorescent staining of lipid droplets. (**I**) Lipodroplet statistical analysis diagram. Data are represented as mean ± SEM of three independent experiments; and ns *p* > 0.05; ****p < 0.0001 vs. the control group

Emerging evidence underscores the critical role of SREBF1 post-translational regulation in governing de novo lipogenesis [27]. Our previous study confirmed that PRMT1 is significantly elevated in patients with liver cancer and is associated with prognosis [28]. Moreover, our multi-parametric target screening identified PRMT1 as a potential CTD or RG3-binding candidate, with mRNA level downregulation after combination therapy (Fig. 2G). The protein level also decreased in CTD/RG3 treated HCC cells (Fig. 5A, B). In addition, molecular docking analyses revealed stable binding of both RG3 and CTD to PRMT1, with calculated binding energies of -8.3 and -5.9 kcal/mol for RG3 and CTD, respectively. Notably, RG3 exhibited a stronger binding affinity for PRMT1 compared to CTD, suggesting a potentially more potent inhibitory effect on PRMT1 function (Fig. 5C). Furthermore, we also performed CETSA to validate this interaction. Treatment with CTD/RG3 compound increased the thermal stability of PRMT1, which was confirmed by the rightward shift of protein bands under increasing temperature conditions (Fig. 5D, E). Taken together, CTD/RG3 compound might directly interact with PRMT1.

To investigate the molecular mechanisms underlying the regulation of the PRMT1-SREBF1 axis by CTD/RG3, rescue experiments were conducted via PRMT1 overexpression in RG3/CTD-treated HCC cells. WB results showed that the SREBF1-related lipid metabolism axis was significantly suppressed in HCC cells subjected to combined RG3 and CTD treatment, whereas PRMT1 overexpression notably attenuated this suppressive effect (Fig. 5F, G). Consistently, Nile red staining analysis revealed that PRMT1 overexpression significantly alleviated the inhibitory impact of the combination therapy on lipid droplet formation (Fig. 5H, I). These findings indicate that PRMT1 may represent a promising target for CTD and RG3 in the regulation of fatty acid synthesis in HCC.

**Fig. 6:**
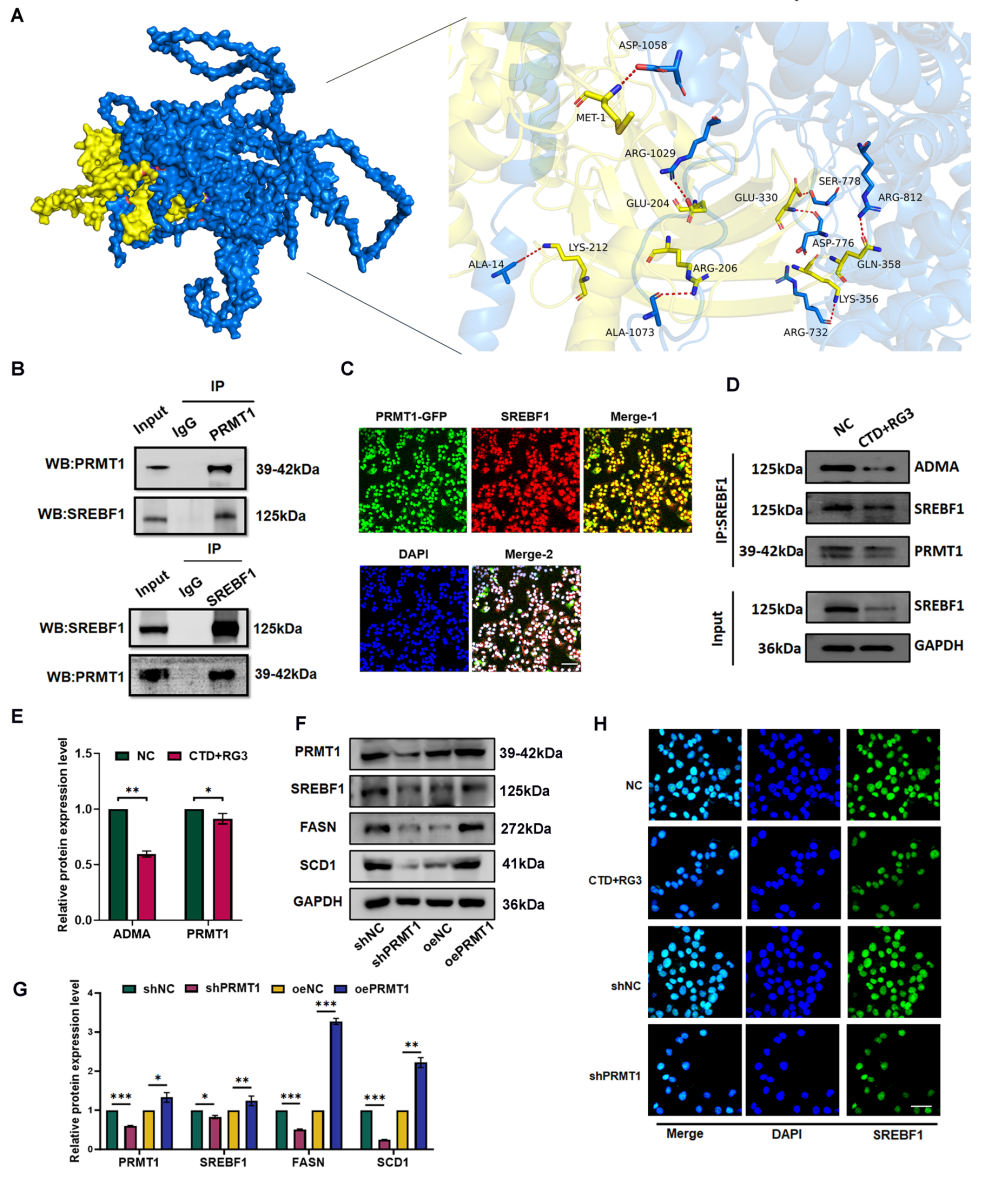
RG3/CTD attenuates SREBF1 methylation modification by PRMT1. (**A**) Simulation of the interaction between proteins PRMT1 and SREBF1. (**B**) Co-IP assay using PRMT1 or SREBF1 antibodies demonstrate the interaction between PRMT1 and SREBF1 in HepG2 cells. (**C**) Immunofluorescence imaging revealed the co-localization of PRMT1 and SREBF1 in HCC cells. The scale bar represents 20 μm; magnification 20×. (**D**, **E**) The methylation level of SREBF1, SREBF1 protein was immunoprecipitated from HCC cells treated with the combination of RG3 and CTD, followed by Western blot analysis to evaluate its interaction with PRMT1 and the ADMA signaling. (**F**, **G**) The protein level of SREBF1, FASN and SCD1 in PRMT1 knockdown and overexpression HepG2 cells. (**G**) Grayscale analysis of protein bands. (**H**) Immunofluorescence results of SREBF1 protein in HepG2 cells treated with the combination of RG3 and CTD, and in PRMT1 knockdown HepG2 cells. The scale bar represents 20 μm; magnification 40×. Data are represented as mean ± SEM of three independent experiments; and ns *p* > 0.05; *p < 0.05; **p < 0.01; ***p < 0.001 vs. the control group

To validate the interaction between PRMT1 and SREBF1, we performed protein interaction structural prediction analysis. The results demonstrated a strong binding affinity between the two proteins, with a binding energy of -15.1 kcal/mol. The binding interface was compact, with complementary surface interactions and stabilizing hydrogen bonds, notably involving arginine residues 732 and 1029, which showed strong interactions (Fig. 6A). Subsequently, Co-IP assays confirmed a direct protein-protein interaction between PRMT1 and SREBF1 in HCC cells (Fig. 6B). Furthermore, immunofluorescence analysis further revealed substantial nuclear colocalization of PRMT1 and SREBF1, suggesting a potential physical interaction between these proteins (Fig. 6C).

Given PRMT1’s function as a methyltransferase, we further evaluated the effects of combined treatment on its enzyme activity-dependent post-translational modifications. Immunoblot analysis of Co-IP complexes demonstrated a significant reduction in SREBF1 methylation levels following CTD/RG3 exposure in HepG2 cells (Fig. 6D, E). We further investigated the effect of PRMT1 on SREBF1 function by examining SREBF1 expression in HCC cells with either PRMT1 overexpression or knockdown. WB analysis revealed that SREBF1 protein levels decreased upon PRMT1 knockdown but increased following PRMT1 overexpression (Fig. 6F, G). Consistent with the changes in SREBF1, the expression of its target genes, such as FASN and SCD1, was upregulated by PRMT1 overexpression and downregulated by its knockdown (Fig. 6F, G). Additionally, the nuclear localization of SREBF1 is not affected in PRMT1-knockdown HCC cells or PRMT1 inhibition through CTD/RG3 treatment (Fig. 6H). Collectively, these data indicate that CTD and RG3 disrupts PRMT1-mediated methylation level of SREBF1, thereby downregulating its expression and disrupting fatty acid synthesis homeostasis.

### RG3 alleviates the hepatotoxicity induced by CTD in mice

To systematically evaluate the hepatoprotective potential of RG3 against CTD-induced liver injury, we employed a dual-model experimental paradigm comprising, including an ectopic HCC cell allograft model, and a CTD-induced acute hepatotoxicity model. Hepatic function was comprehensively assessed through biochemical analysis of liver injury markers and histopathological evaluation.

**Fig. 7:**
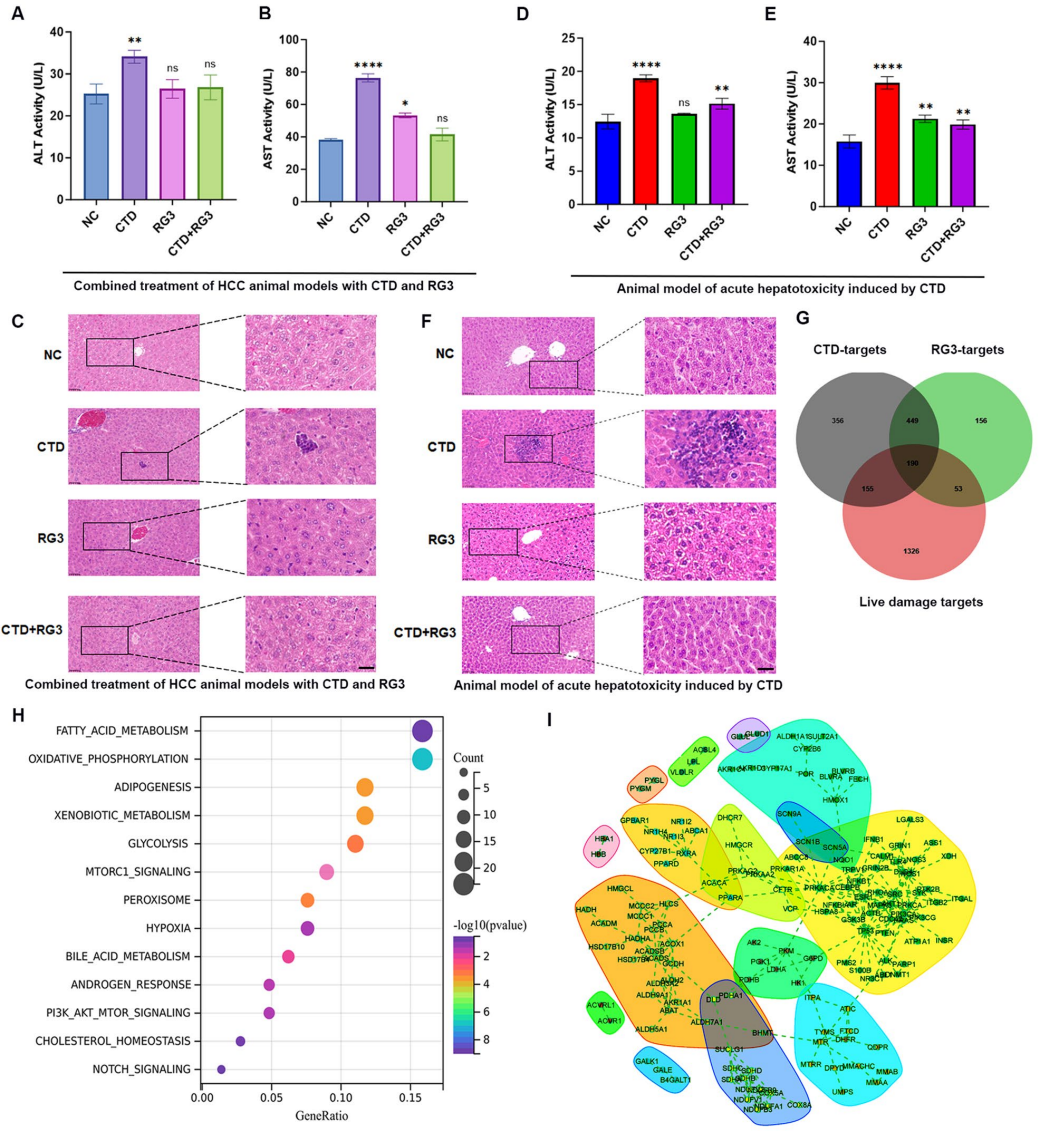
Identification the mechanism of RG3 protection against CTD-induced hepatotoxicity. (**A**, **B**) Effects of administration on serum levels of ALT and AST in mice allograft model with HCC cells. (**C**) Liver sections from mice allograft with HCC cells were stained with H&E. The scale bar represents 20 μm; magnification 40×. (**D**, **E**) Effects of RG3 administration on serum level of ALT and AST in CTD-induced acute hepatotoxicity mice. (**F**) H&E staining of liver sections from mice 14 days after CTD exposure and RG3 intervention in acute hepatotoxicity mice. The scale bar represents 20 μm; magnification 40×. (**G**) Venn diagram of CTD and RG3 bio-active compounds-related targets and liver injury-related targets. (**H**) KEGG enrichment analysis of the relationship between these targets and their associated pathways. (**I**) PPI network diagram of potential targets of RG3 protection against CTD-induced hepatotoxicity. Data are represented as mean ± SEM of three independent experiments; and *p < 0.05; **p < 0.01; ****p < 0.0001 vs. the control group

In the CTD/RG3 co-treated HCC allograft model, the combination regimen demonstrated significant hepatoprotective activity, as evidenced by substantial reductions in CTD-induced elevation of ALT and AST levels compared to CTD monotherapy (Fig. 7A, B). Histopathological evaluation via H&E staining corroborated these biochemical observations, revealing well-preserved hepatic architecture with only scattered inflammatory infiltrates and absence of necrotic foci in the RG3 and CTD combined group. This contrasted sharply with the CTD-treated group, which exhibited diffuse lobular inflammation and focal hepatic necrosis (Fig. 7C).

For the CTD-induced acute hepatotoxicity model, biochemical analysis also revealed a significant elevation of serum ALT and AST levels in CTD-treated mice compared to controls. Notably, concurrent administration of RG3 markedly attenuated these elevations, with ALT and AST levels in the combination group showing significant reduction compared to CTD monotherapy (Fig. 7D, E). HE results demonstrated that CTD-treated livers exhibited disrupted hepatic architecture with prominent hepatocellular ballooning, nuclear pyknosis, and diffuse inflammatory cell infiltration. In contrast, RG3 co-treatment preserved near-normal lobular organization with only mild perivascular inflammation (Fig. 7F). Collectively, these findings demonstrate that RG3 co-administration effectively preserves hepatic function and structural integrity in CTD-treated mice, suggesting its potential as a hepatoprotective adjuvant in CTD-therapy regimens.

### Integrated network analysis to elucidate the protective mechanism of RG3 for CTD-induced hepatotoxicity

**Fig. 8:**
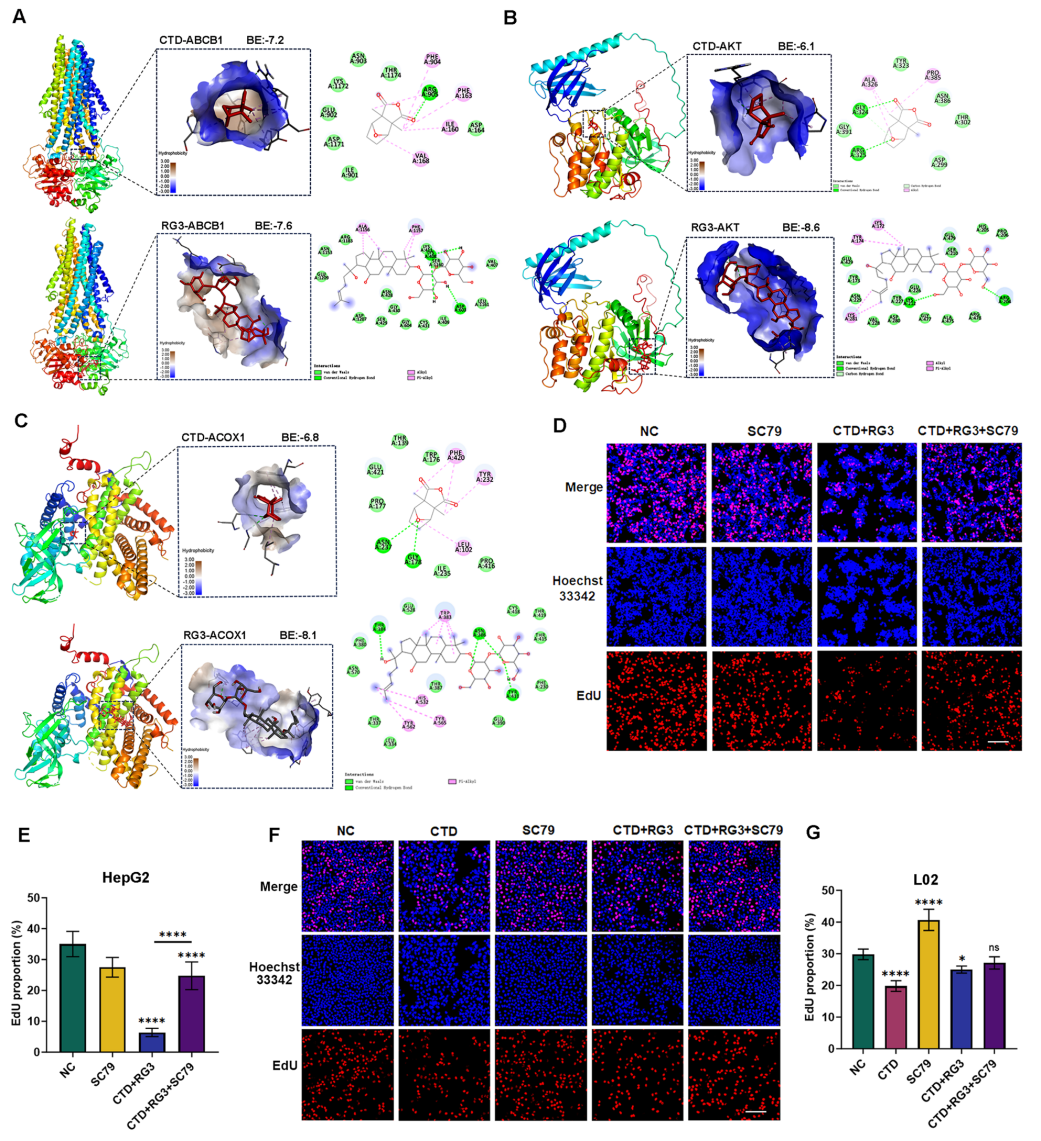
Molecular docking diagram of CTD and RG3 with the key target molecules. (**A–C**) Molecular docking models of (**A**) ABCB1, (**B**) AKT, and (**C**) ACOX1 with CTD or RG3. (**D**, **E**) The AKT activator SC79 rescues CTD/RG3-induced suppression of DNA replication in HepG2 cells, as assessed by EdU assay. (**D**) Representative confocal micrographs, and (**E**) corresponding quantitative analysis. Nuclei were stained with DAPI (blue); EdU-positive proliferating cells are shown in red. (**F**, **G**) Cytotoxicity assessment of the treatments on normal L02 hepatocytes by EdU assay. (**F**) Representative images, and (**G**) quantitative analysis of cell proliferation following treatment with CTD/RG3 in the presence or absence of the AKT activator SC79. The scale bar represents 20 μm; magnification represent 20×. Data are presented as mean ± SEM (n = 3 independent experiments); and ns *p* > 0.05; *p < 0.05; ****p < 0.0001 versus the control group

To clarify the molecular mechanisms that underlie the protective effects of RG3 against CTD-induced hepatotoxicity, we identified 1,724 molecular targets associated with hepatotoxicity from the Comparative Toxicogenomics Database and GeneCards. Subsequently, we conducted an integrative bioinformatics analysis, 190 overlapping targets were identified as potential therapeutic candidates for RG3 in the management of CTD-induced hepatotoxicity (Fig. 7G). Pathway enrichment analyses were further performed and revealed that RG3 exerts pleiotropic regulatory effects through differential modulation of key metabolic and signaling pathways, including fatty acid metabolism, oxidative phosphorylation, xenobiotic metabolism and glycolysis, mTORC1, PI3K and notch signaling pathways (Fig. 7H). Network pharmacology further delineated RG3’s polypharmacological action through a high-confidence PPI network (node score > 0.85, Fig. 7I). Topological analysis identified 10 hub genes (*ABCB1*, *AKT*, *ACOX1*, *ACTB*, *ACACA*, *ESR1*, *GSK3B*, *ACADM*, *DLD*, *NFKB1*) bridging eight functional communities, with AKT/ABCB1 coordinating xenobiotic metabolism-energy homeostasis crosstalk and ACOX1 linking fatty acid degradation to oxidative stress responses. Molecular docking validated these network predictions, demonstrating RG3’s superior binding affinity versus CTD across key targets: ABCB1 (-7.6 vs. -7.2 kcal/mol), AKT (-8.6 vs. -6.1 kcal/mol), and ACOX1 (-8.1 vs. -6.8 kcal/mol) (Fig. 8A-C). The strongest interaction occurred between RG3 and AKT, suggesting this kinase as the primary mediator of RG3’s hepatoprotection.

Given the central role of AKT signaling in metabolism and oxidative stress, along with its high binding affinity for the CTD/RG3 combination, we investigated whether AKT activation could alleviate hepatotoxicity using the AKT activator SC79. EdU assay was performed and revealed that SC79 reversed the suppression of proliferation in HepG2 cells induced by CTD/RG3 (Fig. 8D, E). To further validate the protective role of RG3 against CTD-induced hepatotoxicity, we assessed the cytotoxicity of the combination therapy on normal L02 hepatocytes. The EdU assay revealed that the combination therapy exhibited differential cytotoxicity, showing minimal effects on LO2 cells compared to its significant impact on HCC cells. Moreover, this cytotoxicity induced by CTD was effectively mitigated by RG3 or AKT activation in L02 cells (Fig. 8F, G). Collectively, these findings indicate that RG3 ameliorates CTD-induced liver injury via a multi-target mechanism centered on AKT, which modulates metabolic detoxification, redox homeostasis, and inflammatory signaling.

## Discussion

In recent years, remarkable advances have been achieved in the systemic treatment of HCC, driven largely by the introduction of molecular targeted drugs and immune checkpoint inhibitors. These therapies have markedly improved survival and treatment response in patients with advanced HCC. Nevertheless, the emergence of both primary and acquired resistance remains a major clinical challenge, posing a substantial constraint on long-term therapeutic efficacy. Thus, developing novel combination strategies to enhance the effectiveness of targeted and immunotherapeutic agents represents an urgent unmet need in HCC management.

As an integral component of complementary and alternative medicine, traditional Chinese medicine has demonstrated promising potential in HCC treatment. Its multifaceted mechanisms include inhibiting tumor cell proliferation, inducing apoptosis and autophagy, suppressing metastasis and angiogenesis, as well as modulating antitumor immunity. CTD has been widely utilized in traditional Chinese medicine due to its potent anticancer properties, particularly in HCC. Our previous investigations have systematically characterized CTD’s multimodal mechanisms, including autophagy modulation, EZH2/H3K27me3-dependent cell cycle regulation, and enhanced antitumor immunity [4, 10]. Furthermore, CTD derivative enhances the antitumor activity of sorafenib by inhibiting the IL-6/STAT3 pathway in HCC [29, 30]. Despite these advancements, the precise molecular mechanisms underlying CTD’s anticancer effects remain incompletely understood. Moreover, its clinical application is limited by dose-dependent hepatotoxicity. Therefore, our research focuses on developing strategies to mitigate CTD’s toxicity while enhancing its therapeutic efficacy in HCC treatment.

Previous studies have demonstrated that the Aidi injection formulation, combining CTD with ginsenosides Rc/Rd, exemplifies this paradigm through PP2A activation and UPS inhibition, effectively inducing mitochondrial apoptosis in HCC [2, 31]. Notably, previous studies have explored CTD combinations with anti-angiogenic agents such as bevacizumab, apatinib, and endostar in pancreatic cancer [30]. Building on these findings, we investigated the combination of CTD with RG3, a ginsenoside with demonstrated anticancer properties. Our study is the first to systematically evaluate the therapeutic effect and its molecular mechanism of CTD and RG3 combination in HCC.

As we all know, lipid metabolic reprogramming plays a crucial role in the pathophysiology of liver cancer [32]. Notably, sterol regulatory element-binding protein 1 (SREBP1), encoded by SREBF-1, playing a central role in tumorigenesis, metabolic regulation, metastasis, immune evasion, and therapy resistance [27, 33]. As a master regulator of de novo lipogenesis, SREBP1 represents a promising therapeutic target in HCC. Previous studies have shown that ginsenosides, such as Rh2, can inhibit SREBP1 expression and nuclear translocation, disrupting the SREBP1-FASN axis in non-small cell lung cancer [34]. In our prior work, bioinformatics analysis revealed that CTD target genes are significantly enriched in metabolic pathways, particularly fatty acid metabolism [10]. However, the specific roles of CTD and RG3 in metabolic regulation during cancer treatment remained unclear.

It has been reported that the PI3K/AKT/mTOR pathway is a master regulator of cell growth, proliferation, and survival. PI3K activation enhances SREBF1 nuclear translocation and transcriptional activity, upregulating enzymes like FASN and SCD1 [35]. In this study, our findings revealed that RG3 enhances CTD’s inhibitory effects on fatty acid synthesis through a synergistic mechanism, and establishes a novel therapeutic paradigm involving dual-pathway regulation of lipid metabolism. Our current work expands the understanding of its metabolic regulatory capacity in HCC. Mechanistically, the RG3-CTD combination demonstrates multi-level regulatory effects on lipid metabolism networks. CTD and RG3 could be targeted to bind to PI3K protein, this coordinated inhibition of PI3K/AKT/mTOR signaling leads to concerted downregulation of SREBF1 expression and subsequent suppression of its target lipogenic enzymes. Therefore, combination therapy could effectively suppress the PI3K-AKT-mTOR pathway to block abnormal fatty acid synthesis in liver cancer cells.

**Fig. 9:**
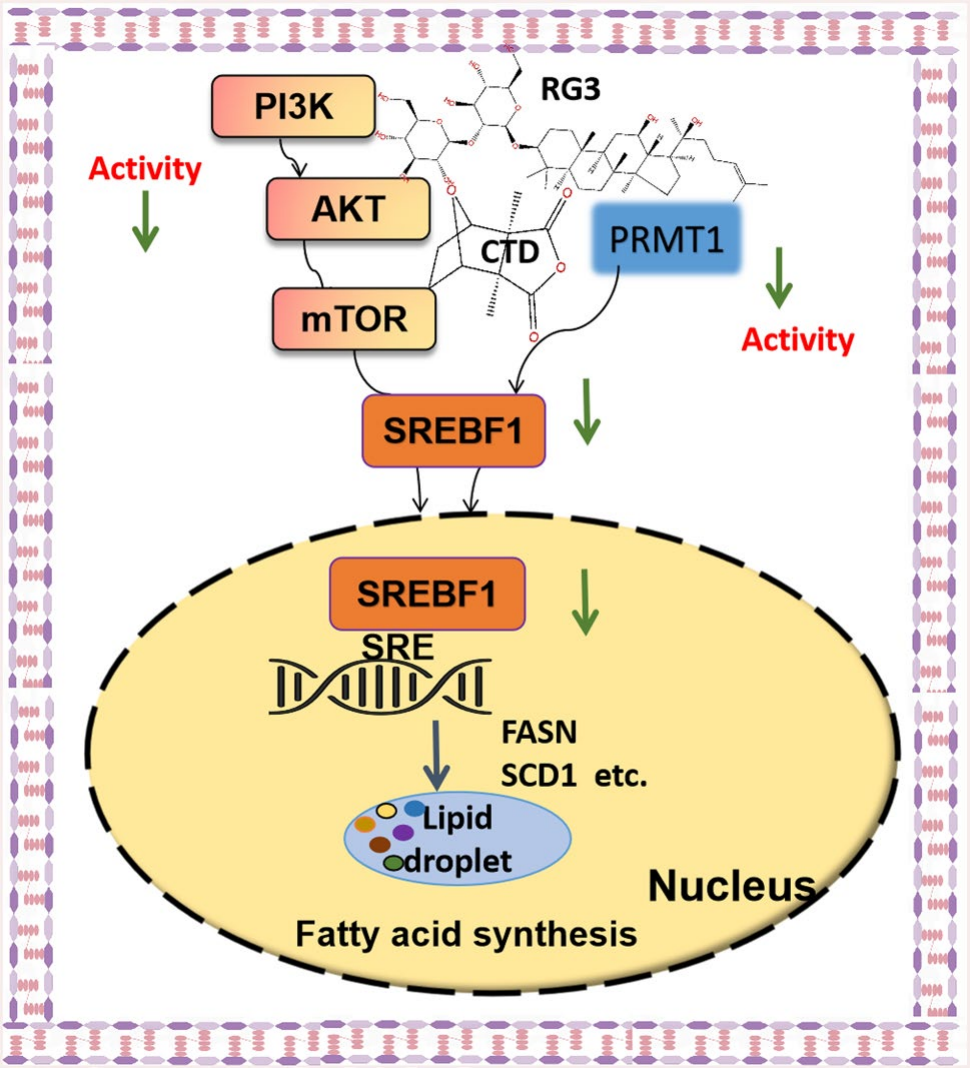
Mechanism model diagram of the RG3-CTD complex synergistically suppresses lipogenesis via a dual-pronged mechanistic strategy in HCC treatment

PRMT1, a critical epigenetic modulator in oncogenesis, demonstrates broad substrate specificity through its protein arginine methyltransferase activity. Previous studies have established its role in post-translational modification of metabolic enzymes including PFKFB3, PKM2, and PHGDH, which collectively promote de novo fatty acid biosynthesis in breast cancer models [36]. We also revealed PRMT1’s dual oncogenic functions in HCC, involving both immune microenvironment modulation and lipid metabolic reprogramming [37]. In this study, we find that PRMT1 directly interacts with SREBF1, and exhibits specific binding affinities for CTD and RG3. Critically, combination therapy downregulates PRMT1 expression, thereby disrupting the SREBF1 pathway and fatty acid synthesis. In conclusion, we propose that PRMT1 methylates SREBF1 arginine residues, enhancing its stability/activity to amplify lipogenic gene expression, thereby promoting the progression of HCC. Targeted binding of CTD/RG3 to PRMT1 likely impairs its methyltransferase activity, reducing SREBF1 methylation level and suppressing downstream signaling (Fig. 9).

Beyond enhancing CTD’s antitumor efficacy, significant efforts have been directed toward understanding and mitigating its hepatotoxicity. Emerging evidence suggests that CTD-induced liver injury involves complex apoptotic and autophagic processes [38]. The combination of CTD with hepatoprotective agents, such as ginsenosides, has shown promise in reducing toxicity while maintaining therapeutic efficacy. Aidi injection, a formulation containing extracts from *Mylabris* phalerata, *Astragalus* membranaceus, and *Panax* ginseng, has demonstrated clinical utility as an adjuvant in cancer therapy, highlighting the potential of ginsenosides to counteract CTD’s toxicity [39]. Consistent with these findings, our study confirms that RG3 alleviates CTD-induced liver injury through multifaceted mechanisms, including modulation of the PI3K/AKT, Nrf2, SIRT1/AMPK, and ERK/JNK pathways [15–18, 40].

To further elucidate the protective mechanisms of RG3 against CTD-induced hepatotoxicity, we employed a network pharmacology approach. In recent years, multi-omics technologies have rapidly advanced molecular diagnosis and drug discovery [41]. Integrated with principles from systems biology, network pharmacology has emerged as a powerful tool for predicting the mechanistic basis of TCM therapeutics [42]. This approach holds significant promise for improving clinical diagnosis, prognosis, and treatment strategies in oncology [43]. Currently, transcriptomic and proteomic methodologies are being widely applied in TCM research [43, 44]. In this study, we combined RNA-seq and network pharmacology data to elucidate the molecular targets and mechanisms of action following the co-administration of CTD and RG3 in HCC therapy.

Through rigorous analysis, we identified several critical targets associated with liver injury and constructed a comprehensive protein-protein interaction network. Functional enrichment analysis revealed significant associations with metabolic regulation, glycolysis, fatty acid metabolism, oxidative phosphorylation, and key oncogenic signaling pathways such as mTOR, PI3K, AKT and hypoxia. Structure-based virtual screening identified AKT as the prime molecular target with ultra-high binding affinity with RG3, suggesting that RG3 may selectively target AKT and exert its protective effects against CTD-induced hepatotoxicity by inhibiting the AKT signaling pathway. Taken together, these findings not only deepen our understanding of RG3’s hepatoprotective effects but also provide potential therapeutic targets for mitigating CTD-induced liver injury.

The PI3K/AKT pathway, known for its role in metabolic regulation, also contributes significantly to drug resistance, which remains a major clinical challenge driven by multifactorial mechanisms, including aberrant activation of this pathway [45]. Many studies have demonstrated that ginsenoside RG3 synergizes with various anticancer therapies, such as radiotherapy, chemotherapy, and targeted agents, enhancing their antitumor efficacy [46]. For example, in gastric cancer, RG3 mitigates cisplatin resistance via upregulation of miRNA-429 and suppression of SOX3 and the PI3K/Akt/mTOR axis [47]. In this study, we found that the combination treatment inhibited lipogenesis through modulation of the PI3K/AKT/mTOR pathway, thereby suppressing HCC progression. We speculate that combination therapy containing RG3 may be one of the effective strategies for overcoming drug resistance and restoring sensitivity to standard treatment in HCC. Future studies will focus on elucidating the mechanistic synergy between RG3 and CTD in reversing therapeutic resistance.

In conclusion, this study reveals that the synergistic combination of RG3 and CTD suppresses HCC progression through dual inhibition of SREBF1-mediated lipogenesis via PI3K/AKT/mTOR signaling and PRMT1-dependent post-translational regulation. Crucially, this strategy enhances antitumor efficacy while reducing CTD-induced hepatotoxicity, addressing key limitations of traditional monotherapy. Mechanistic insights into lipid metabolism targeting provide a framework for optimizing natural product-based therapies. Despite promising preclinical results, future work requires validation in orthotopic HCC models and clinical trials to assess tumor microenvironment interactions and therapeutic index refinement. The findings establish a paradigm for developing safer, multi-targeted phytochemical combinations in oncology.

## Supplementary Information

Below is the link to the electronic supplementary material.

Supplementary Material 1

Supplementary Material 2

## Data Availability

All data could obtain from the corresponding authors.
