# Supplementary figures and images for: Ginsenoside RG3 and cantharidin synergistically suppress the progression of hepatocellular carcinoma via targeting the PRMT1-SREBF1 axis-mediated lipid metabolism
Source: J Transl Med. 2026 Jan 7;24:78. doi: 10.1186/s12967-025-07550-8 (PMC12811908; doi:10.1186/s12967-025-07550-8)

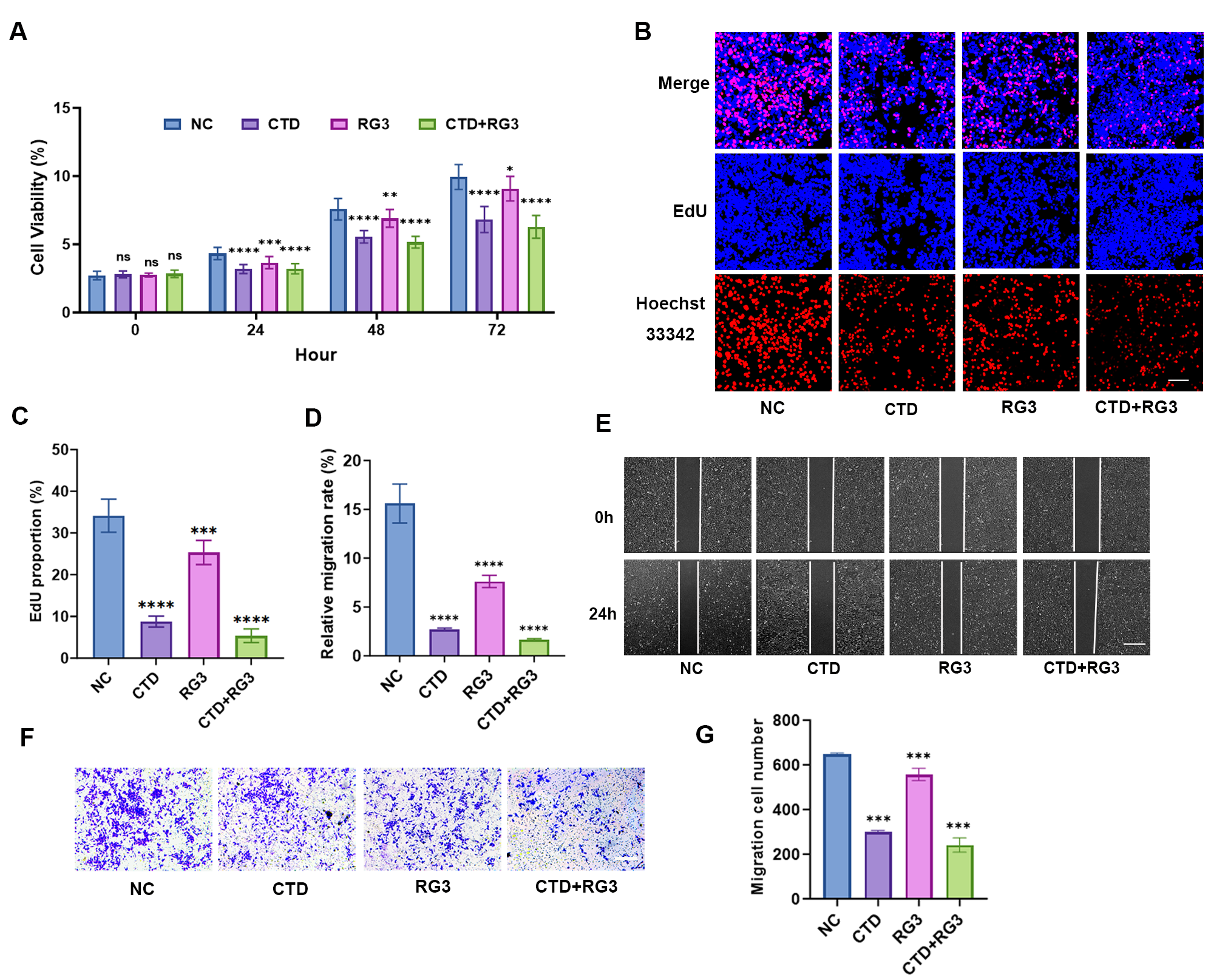

Supplement: Supplementary file 1 — Supplementary Material 1 [file 12967_2025_7550_MOESM1_ESM.tif]

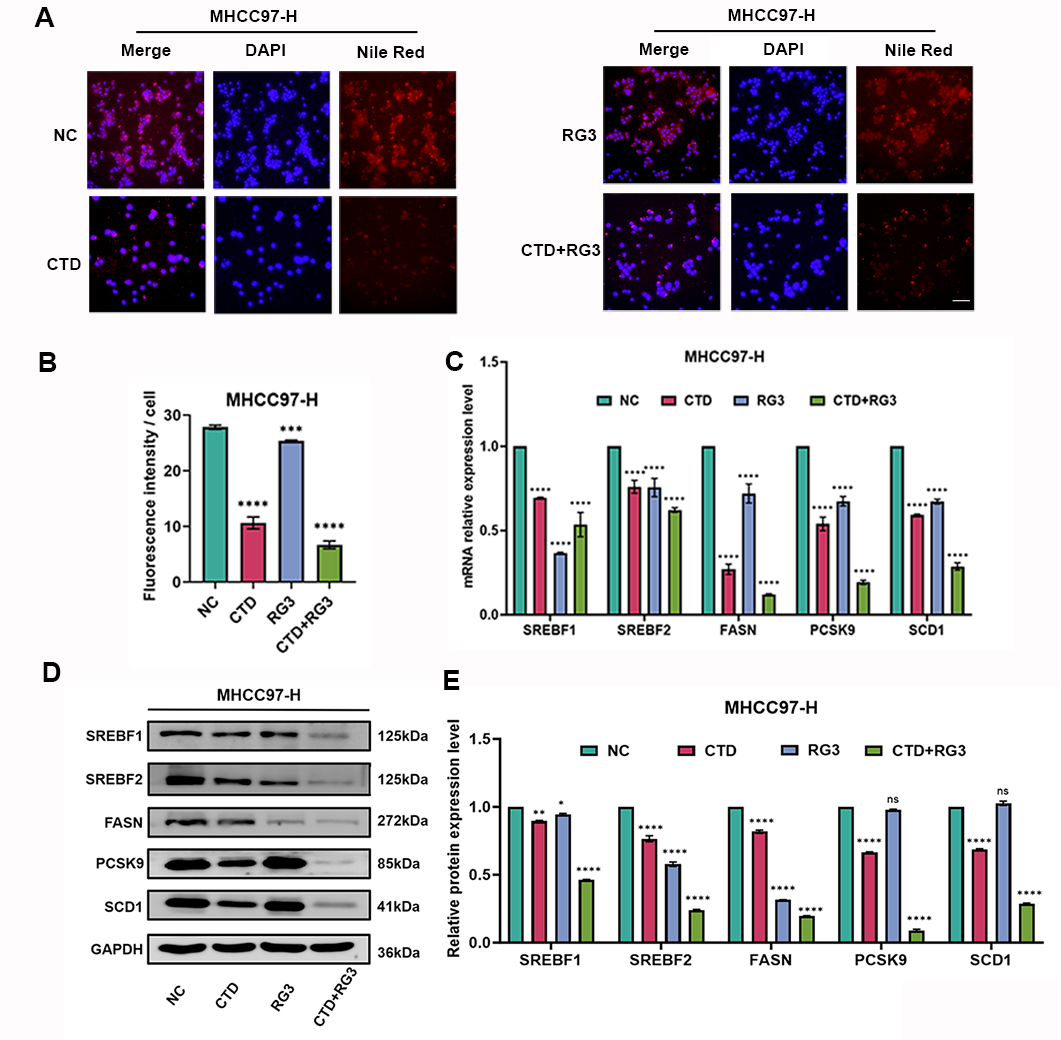

Supplement: Supplementary file 2 — Supplementary Material 2 [file 12967_2025_7550_MOESM2_ESM.tif]
